# Visual Triggers of Skin Picking Episodes: An Experimental Study in Self-Reported Skin Picking Disorder and Atopic Dermatitis

**DOI:** 10.32872/cpe.v2i4.2931

**Published:** 2020-12-23

**Authors:** Linda Marlen Mehrmann, Alice Urban, Alexander Leopold Gerlach

**Affiliations:** a Institute of Clinical Psychology and Psychotherapy, University of Cologne, Cologne, Germany; Philipps-University of Marburg, Marburg, Germany

**Keywords:** skin picking, excoriation disorder, body-focused repetitive behaviors, contagious itch, visual stimuli

## Abstract

**Background:**

Skin Picking Disorder (SPD) is a new diagnosis with limited information available about triggers of picking episodes. Itch can be induced via audio-visual stimuli and the effect of contagious itch is stronger for those affected by atopic dermatitis. We examined if picking-related visual stimuli can trigger the urge to pick skin in self-reported SPD. We compared itch and the urge to pick in a sample of AD and/or SPD-affected to controls without either.

**Method:**

Urge to pick skin and/or scratch when viewing 24 itch-related, picking-related or neutral online pictures was assessed in adult females, who self-report skin-picking (SPD-only, n = 147) and/or atopic dermatitis (AD-only, n = 47; AD+SPD, n = 46) as well as in skin healthy controls (HC, n = 361).

**Results:**

All participants reported a stronger urge to pick for picking-related pictures compared to neutral content (F[1, 597] = 533.96, p < .001, ηp2 = .472) and more itch for itch-related pictures compared to neutral stimuli (F[1, 597] = 518.73, p < .001, ηp2 = .465). SPD-all (SPD-only & AD+SPD) reported stronger urges to pick for picking-related vs. other stimuli compared to the AD-only and HC group (p < .001, ηp2 = .047). Likewise, AD-all (AD-only & AD+SPD) reported significantly stronger itching for itch-related vs. other stimuli compared to SPD-only and HC (p = .001, ηp2 = .019).

**Conclusions:**

Analog to visual provocation of itch, the urge to pick can be triggered by visual stimuli. Treatments for SPD and AD may profit from addressing visual stimuli.

## Abstract (highlights)

- Contagious itch can be induced with visual stimuli (videos or static pictures).
- This effect is especially strong in individuals self-reporting atopic dermatitis.
- The urge to pick can also be induced with visual stimuli (picking-related pictures).
- This effect is especially strong in individuals self-reporting skin picking disorder.
- Treatment for skin picking disorder may profit from addressing visual stimuli.

Skin Picking disorder (SPD) has recently been included as official diagnosis in the Diagnostic and Statistical Manual of Mental Disorders (DSM). DSM-5 characterizes SPD as recurrent skin picking resulting in lesions of the skin and repeated attempts to decrease or stop this behavior. Additionally, for a diagnosis of skin picking disorder, skin picking must cause clinically significant distress or impairment in important areas of functioning (American Psychiatric Association, 2013). Many people indulge in picking behavior from time to time, however, people with SPD feel a strong urge to manipulate their skin and feel unable to resist this urge or to stop (American Psychiatric Association, 2013).

Clinical experience suggests that skin picking episodes can be triggered in various different ways (Mansueto et al., 1997; Neziroglu et al., 2008). However, mostly self-report studies of triggers for skin picking episodes have been published. In a clinical sample emotional triggers such as general anxiety, general stress, interpersonal rejection, a sense of emptiness, and teasing were reported (Neziroglu et al., 2008). In terms of visual stimuli, skin imperfections were most commonly mentioned (80%), including pimples, scabs, scars, and mosquito bites. Regarding somatosensory triggers, itchiness (40%), the feeling of something being underneath the skin surface (32%), and a “right feeling” sensation (40%) were described. The most common environmental triggers were looking in the mirror and checking one’s skin (52%; Neziroglu et al., 2008). In a German nonclinical sample (Bohne et al., 2002; *N* = 133), students reported cutaneous triggers to be pimples (93.2%), insect bites (63.9%), scabs (57.1%), itching (45.9%), inflammation (34.6%), warts (13.5%), healthy skin (18.0%), moles and scars (9.8%). Participants with SPD reported the feel (55%) and sight (26.7%) of the skin as the most common triggers to picking behavior (Odlaug & Grant, 2008). Finally, Houghton et al. (2018) investigated sensory processing in people affected by body-focused repetitive behaviors (BFRBs; e.g., hairpulling, skin picking, nail biting) via the Adult/Adolescent Sensory Profile (Brown et al., 2001). Participants with clinical BFRBs reported increased sensory sensitivity including visual stimuli compared to subclinical BFRBs and healthy controls. In summary, many of these triggers indicate visual perception of one’s skin (e.g., when looking in the mirror) to be one of various factors within the cycle of urge to pick and picking behavior.

One fMRI study examined visual symptom provocation in SPD (Schienle, Ubel, & Wabnegger, 2018). For pictures with skin irregularities, disgust, tension and urge to pick ratings were significantly higher in the SPD-group. However, the same was true regarding disgust and urge to pick for pictures without skin irregularities. Furthermore, when looking at skin imperfections, SPD-patients showed greater activation in the left insula and in the amygdala with stronger insula-putamen coupling compared to matched controls. These brain regions are linked to visual disgust elicitation, process salience and the affective significance of stimuli.

Whereas experimental studies examining mechanisms underlying the urge to pick in SPD are mostly lacking, some exist for pruritus, especially pruritus associated with atopic dermatitis (AD). AD presents several similarities with SPD. AD patients suffer from a cutaneous hyperreactivity to environmental triggers resulting in a chronic inflammatory skin disease (Leung, 2013). Pruritus is the cardinal symptom of AD provoking the desire to scratch for relief from this unpleasant sensation but leading to skin damage and other negative consequences (Mochizuki et al., 2014; Ständer & Steinhoff, 2002). However, the mechanical stimulation of the skin may provoke inflammation, which again exacerbates itch (itch-scratch-cycle; Mochizuki et al., 2017). Due to its negative impact on quality of life, most patients measure the severity of their AD by intensity of pruritus rather than visible skin damage (Ständer & Steinhoff, 2002). Against this background, Verhoeven et al. (2008) proposed a biopsychosocial model of itch in patients with chronic skin diseases: internal vulnerability factors (e.g., personality) interact with external environmental factors (e.g., stressors). Meanwhile, cognitive (e.g., illness cognitions), behavioral, and social factors are mediating and/or moderating factors to trigger a skin disease and enhance symptoms of itch. Contagious itch (CI) can therefore be a cognitive psychological factor causal in pathological itch (Verhoeven et al., 2008).

Itch sensations can be evoked through mechanical, electrical, thermal and chemical stimulation of free nerve endings in the skin (Leknes et al., 2007; Murota & Katayama, 2017). Apart from methods manipulating the skin to induce itch (e.g., histamine and allergen solutions), non-skin-manipulating methods also lead to itch sensations (Leknes et al., 2007). For example, itch can be induced with audio-visual stimuli. Niemeier, Kupfer, and Gieler (2000) held two different lectures (“itch lecture”, “relaxation lecture”) for participants with and without self-reported skin disease. Self-reported itch sensation after the lecture as well as the number of scratch movements during the “itch lecture” (slides with pictures of fleas, allergic reactions etc.) were significantly higher compared to the “relaxation lecture”. However, there was only a trend with regard to the experience of itching sensations when comparing participants with and without skin conditions. Ogden and Zoukas (2009) replicated these results with college students without assessing skin conditions using purely visual stimulation (e.g., videos of lice, person scratching head) without audio. In 2011, Papoiu et al. investigated whether exposure to visual cues of itch (5-minute video of people scratching their left forearm vs. people sitting idle) can induce or intensify itch in AD patients and healthy controls. Itch intensity increased slightly in healthy volunteers and significantly in AD patients. The latter also scratched more frequently while watching the itch video. Schut et al. (2015) identified depression as an additional significant predictor of induced itch. Furthermore, agreeableness and public self-consciousness were significant predictors of scratching in AD-patients. Palani et al. (2018) asked healthy participants to watch videos picturing a demonstrator scratching in four body regions with and without sound and a control video with neutral content. Results showed CI to be body-region dependent, with the craniofacial region being the predominant site for participants to experience itching sensations after watching the video compared to arm, chest, and back.

These studies on CI used a lecture or video material to induce itch. Lloyd et al. (2013) tested whether static images (i.e., visual cues alone) were able to induce CI. They used neutral (e.g., butterflies or healthy skin) or itch-related pictures (e.g., fleas or skin conditions). Healthy participants reported higher itch intensity for itch-related pictures compared to neutral pictures, and scratching frequency when viewing the pictures was significantly higher for itch-related pictures. Furthermore, more scratch movements for the “skin response” picture type (e.g., scratching an insect bite) were found. Lloyd et al. (2017) tested whether a history of pruritic skin conditions moderates the CI effect when looking at static pictures. Itch-related pictures again caused higher self-itch. Furthermore, participants with a history of pruritic skin conditions gave higher self-itch ratings when viewing “skin response”-images. In summary, somatosensory perception in the absence of somatosensory stimulation (i.e., CI) can be induced via the presentation of sounds, pictures or videos (Schut et al., 2015) and is enhanced in individuals suffering from chronic itch-related skin conditions.

In the present study, we test if this type of effect (i.e., CI) can be replicated with other types of stimuli and reactions – specifically, with visual stimuli triggering the urge to pick one’s skin. We investigated whether picking-related visual stimuli compared to other stimuli (itch-related, neutral) trigger the urge to pick in SPD-affected compared to persons without SPD. Comparably, we tested, whether itch-related visual stimuli compared to other stimuli (picking-related, neutral) trigger itch sensations in AD patients compared to participants without AD. Our investigation could experimentally present a pathological mechanism previously mainly self-reported as a relevant trigger for skin picking episodes in SPD.

## Method

### Design

In a quasi-experimental study (stimulus type [3] ☓ group [4]), data was collected online with Enterprise Feedback Suite Survey. Following the guidelines of the German Psychological Society, all participants provided written informed consent prior to participation.

#### Procedure

The survey was disseminated in several recruitment waves, among others the newsletter of a German self-help group for skin picking and in forums focusing on AD and pruritus. After the initial data collection of SP affected individuals (*N* = 307; SPD: 74%, AD: 4%, HC: 22%, male gender was substantially underrepresented (9.5%). Given that it was unlikely that we would be able to recruit a sufficient number of male participants, we thenceforth exclusively targeted female AD-patients and healthy controls in the following recruitment waves. After an introductory text and informed consent (following the ethical guidelines of the German Psychological Society, see Appendix B5), sociodemographic information was assessed. Derived from DSM-5 criteria for SPD a three-question (criteria A-C) screening was conducted (KSSP, *N* = 601, α = .86; Mehrmann, Hunger, & Gerlach, 2017). As soon as participants reported feeling impaired due to SPD, they were allocated to the SPD group. Additionally, participants were asked about skin diseases (e.g., AD, psoriasis, lice). When answering positive regarding AD (current symptoms or symptoms in the past three months), they were allocated to the AD-group.

### Materials

#### Visual Stimuli

Following a short explanation to German synonyms and difference between picking and scratching (see Appendix B4), every participant looked at 24 visual stimuli (500x759 pixel) in random order (see additional information in Appendix B1). The stimuli consisted of 24 static images of human skin sourced from Google images and one photo specifically taken for this project. Similar to the stimulus material used by Lloyd et al. (2017) eight pictures represented one of three stimulus types each: (1) picking-related images depicting pimples, scabs, or loosening skin flakes, (2) itch-related images with skin conditions (e.g., eczema, mosquito bites), and (3) neutral images with pictures of intact, healthy skin. For each stimulus type, two images of four different body parts (head, torso, arm/hand, leg) were included. After looking at each stimulus a minimum time of three seconds the participants could click to the next page and answer four questions on a 5-point Likert-type scale (0 = not at all, 4 = very strong): “How itchy do you feel?” (itch-self), “How itchy do you think the person in the picture feels?” (itch-other), “How strong is your urge to pick (not scratch)?” (urge-to-pick-self), “How strong do you think is the urge to pick (not scratch) of the person in the picture?” (urge-to-pick-other). Given that participants were free to look at the pictures as long as they wished, we checked for differences between viewing times. However, there was no main effect of viewing times for stimulus-type, Pillai’s trace *V* = .002, *F*(2, 596) = .69, *ns*, $\eta_{p}^{2}$ = .002; no effect for group, *F*(3, 597) = 1.93, *ns*, $\eta_{p}^{2}$ = .01 and no interaction effect for stimulus-type ☓ group, Pillai’s trace *V* = .004, *F*(6, 1194) = .42, *ns*, $\eta_{p}^{2}$ = .002.

#### Questionnaires

Several questionnaires were used to assess AD and SPD as well as general measures of psychopathology. AD or SPD specific questionnaires were only presented if participants screened positive for one or both of them.

##### mSPS-D

The modified Skin Picking Scale, German version (Mehrmann et al., 2017), is a translated and adapted version of the Skin Picking scale by Keuthen, Wilhelm, et al. (2001; Snorrason et al., 2012) and the Massachusetts General Hospital (MGH) Hairpulling Scale (Keuthen et al., 1995). Nine items measure frequency and intensity of picking as well as impairment due to skin picking on a 5-point Likert-type scale. Scores can range from zero to 36 (*n* = 515, α = .95). Currently, there is no clinical cut-off score for the German version available.

##### mSPS-D-AD

To use a similar scale to explore the AD-sample, we modified the mSPS-D by exchanging the words “Picking” and “skin picking” with “scratching” and “atopic dermatitis” (*n* = 105, α = .89).

##### SPIS-D

The Skin Picking Impact Scale by Keuthen, Deckersbach, et al. (2001) was translated into German (Mehrmann et al., 2017). A short version with four items (Snorrason et al., 2013) measures psychosocial impairment due to skin picking on a 5-point-Likert-type scale (*n* = 515, α = .97). For the original version, Keuthen, Deckersbach, et al. (2001) propose a score ≥ 7 to determine clinical impairment.

##### SPIS-D-AD

Participants with AD symptoms answered an AD-adapted version (see above) of the SPIS-D items for psychosocial impairment (*n* = 105, α = .89).

##### AD-scale

AD-affected answered a three-question scale on feeling itchy and actual scratching during the last two weeks as well as impairment due to AD via a 5-point Likert-type scale (Stangier, Gieler, & Ehlers, 2013; *n* = 105, α = .83).

##### BSI-18

The German short version of the Brief Symptom Inventory (Franke, 2000; Spitzer et al., 2011) is a self-report symptom scale assessing levels of psychological distress. Eighteen items with a 5-point Likert-type scale result in a global severity scale (GSI) ranging between 0 and 72 (*n* = 598, α [GSI] = .91).

### Sample

Primary inclusion criteria were consent to study participation, age > 18 years, female gender and completion of the online survey. Altogether, 764 out of 1,406 participants met all primary inclusion criteria. 163 participants were excluded during data processing, because they reported other skin conditions during the last three months, with symptoms that could be confounded with itch or the urge to pick, e.g. mycosis pedis, parasites. The final data set contained 601 participants. The four groups were represented as followed: *n* (AD_only_) = 47 (7.8%), *n* (SPD_only_) = 147 (24.5%), *n* (AD+SPD) = 46 (7.7%), *n* (HC) = 361 (60.0%). Post hoc tests showed the AD_only_-group to be significantly older than the SPD_only_-group (-5.05, 95%-CI [-9.77, -.34]).There was only a small negative correlation between age and the perception of itch (*r* = -.11, *p* = .04), or the urge to pick (*r* = -.16, *p* = .003) for the HC-group. See Table 1 for questionnaire-scores (see additional information in Appendix B2).

**Table 1:** Descriptive Statistics for All Questionnaires With Univariate Analysis

Questionnaire	AD_only_ (*n* = 47)		SPD_only_ (*n* = 147)		AD+SPD (*n* = 46)		HC (*n* = 361)		*F*	*df1, df2*	$\eta_{p}^{2}$
	*M*	*SD*	*M*	*SD*	*M*	*SD*	*M*	*SD*			
Age	34.32	13.17	29.27	9.63	29.91	11.27	31.55	10.58	3.30*	3, 597	.016
mSPS-D	–	–	20.60	5.48	20.41	5.10	5.38	4.66^a^	565.01**	2, 509	.689
SPIS-D	–	–	10.59	4.19	6.85	4.60	0.74	1.88^a^	554.53**	2, 509	.685
mSPS-D-AD	17.21	7.24	–	–	21.57	5.56	–	–	10.54*	1, 91	.104
SPIS-D-AD	6.36	4.96	–	–	8.41	4.55	–	–	4.31*	1, 91	.045
BSI-18	13.81	9.93	18.41	11.64 ^b^	16.26	10.29	8.14	9.41	40.07**	3, 595	.168

*Note.* SPD_only_ = Skin Picking Disorder; AD_only_ = Atopic Dermatitis; AD+SPD = Atopic Dermatitis and Skin Picking Disorder; HC = Healthy control; mSPS-D = modified Skin Picking Scale, German version; SPIS-D = Skin Picking Impact Scale, German version; mSPS-D-AD = modified SPS-D for AD; SPIS-D-AD = modified SPIS-D for AD; BSI-18 = German short version of the Brief Symptom Inventory.

^a^*n* = 319. ^b^*n* = 145.

*p < .05, two-tailed. **p < .001, two-tailed.

### Analysis

All participants were allocated to one of four groups (AD_only_, SPD_only_, AD+SPD, HC). Sociodemographic characteristics and questionnaires were tested using an ANOVA and we used the Bonferroni method as provided by SPSS to adjust for multiple comparisons in the post-hoc tests. In a repeated measures MANOVA, itch-other and urge-to-pick-other ratings were analyzed for stimulus type (itch-related, picking-related, neutral), followed by univariate ANOVAs and planned contrasts. In a repeated measures MANOVA itch-self and urge-to-pick-self ratings were analyzed for stimulus type (3) ☓ group (4) with separate univariate ANOVAs and planned contrasts (see additional information in Appendix B3). When sphericity was violated, the Greenhouse–Geisser adjustment was used.

## Results

### Manipulation Check (Urge-To-Pick-Other and Itch-Other Ratings)

A MANOVA revealed a significant effect of urge-to-pick-other and itch-other ratings for stimulus type, Pillai’s trace *V* = .92, *F*(4, 597) = 1714.56, *p* < .001, $\eta_{p}^{2}$ = .920, indicating the experience of itch and the urge to pick varied based on picture content.

#### Urge-To-Pick-Other

In the univariate ANOVA a significant effect for stimulus type was revealed, *F*(1.89, 1135.54) = 1027.02, *p* < .001, $\eta_{p}^{2}$ = .631. Urge-to-pick-other ratings were significantly higher for picking-related stimuli (*M* = 1.64, *SD* = .77) than for neutral stimuli (*M* = .27, *SD* = .37), *F*(1, 600) =2344.64, *p* < .001, $\eta_{p}^{2}$ = .796. Urge-to-pick-other ratings were also significantly higher for picking-related stimuli than for itch-related stimuli (*M* = 1.41, *SD* = .95), *F*(1, 597) = 11.24, *p* = .001, $\eta_{p}^{2}$ = .018.

#### Itch-Other

In the univariate ANOVA a significant effect for stimulus type was revealed *F*(1.97, 1181.05) = 3465.76, *p* < .001, $\eta_{p}^{2}$ = .852. Itch-other ratings were significantly higher for itch-related stimuli (*M* = 2.34, *SD* = .67) than for neutral stimuli (*M* = .31, *SD* = .38), *F*(1, 600) = 6543.65, *p* < .001, $\eta_{p}^{2}$ = .916. Itch-other ratings were significantly higher for itch-related stimuli than for picking-related stimuli (*M* = 1.11, *SD* = .66), *F*(1, 597) = 1186.43, *p* < .001, $\eta_{p}^{2}$ = .665.

### MANOVA (Stimulus Type ☓ Group; Urge-To-Pick-Self and Itch-Self Ratings)

The MANOVA revealed a significant main effect for group (Pillai’s trace *V* = .26, *F*[6, 1194] = 29.41, *p* < .001, $\eta_{p}^{2}$ = .129), a significant main effect for stimulus type (Pillai’s trace *V* = .53, *F*[4, 594] = 169.78, *p* < .001, $\eta_{p}^{2}$ = .533), and a significant interaction effect for stimulus type ☓ group (Pillai’s trace *V* = .25, *F*[12, 1788] = 13.41, *p* < .001, $\eta_{p}^{2}$ = .083).

#### Urge-To-Pick-Self Ratings

**Figure 1:**
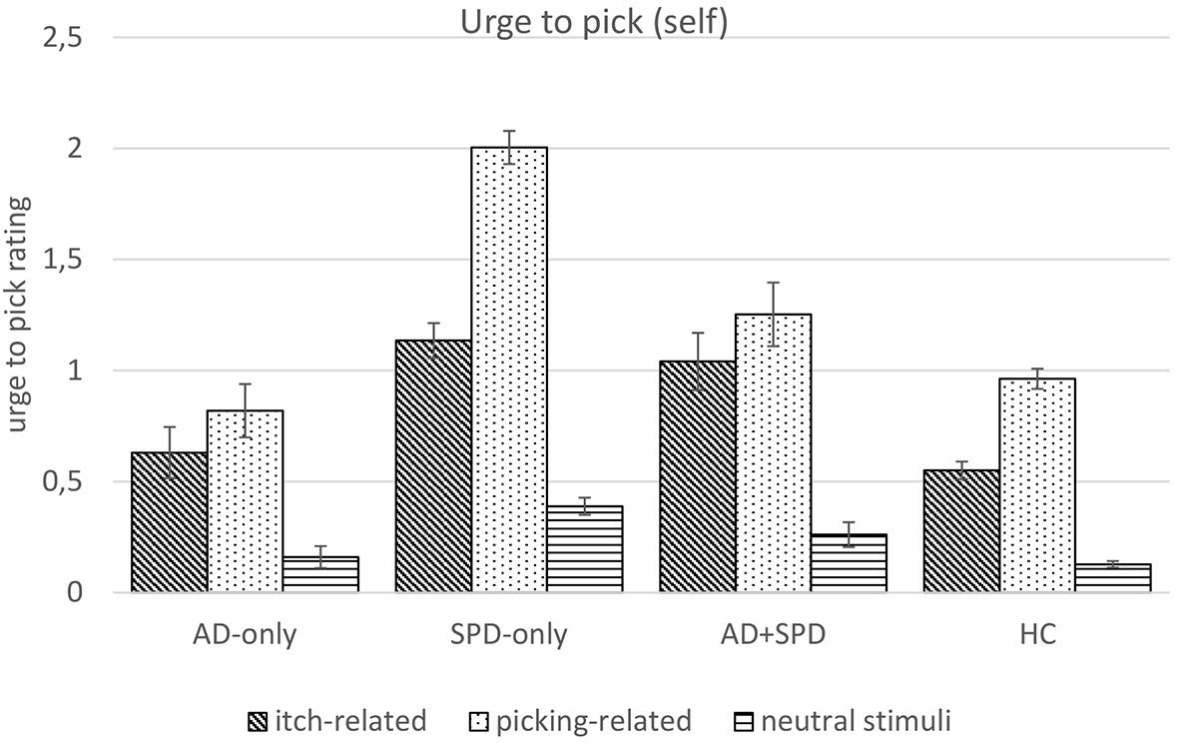
Experienced Urge to Pick (0-4) by Group and Type of Stimulus *Note.* SPD_only_ = Skin Picking Disorder; AD_only_ = Atopic Dermatitis; AD+SPD = Atopic Dermatitis and Skin Picking Disorder; HC = Healthy control. Error bars show standard errors.

Univariate follow-up analyses of urge-to-pick-self ratings again found a significant main effect for stimulus type, *F*(1.96, 1172.76) = 304.54, *p* < .001, $\eta_{p}^{2}$ = .338, and for group, *F*(3, 597) = 42.47, *p* < .001, $\eta_{p}^{2}$ = .176. Additionally, there was a significant interaction effect for stimulus type ☓ group, *F*(5.89, 1172.76) = 24.21, *p* < .001, $\eta_{p}^{2}$ = .108 (see Figure 1; additional tables on urge-to-pick-self and itch-self ratings in Appendix A**).**

All participants experienced a stronger urge to pick when looking at picking-related compared to neutral stimuli, *F*(1, 597) = 533.96, *p* < .001, $\eta_{p}^{2}$ = .472. They also reported a stronger urge to pick when looking at picking-related compared to itch-related stimuli, *F*(1, 597) = 112.41, *p* < .001, $\eta_{p}^{2}$ = .158 and when looking at itch-related compared to neutral stimuli, *F*(1, 597) = 216.11, *p* < .001, $\eta_{p}^{2}$ = .266.

To check whether participants with SPD reported a stronger urge to pick for picking-related stimuli compared to other stimuli, we compared this difference in SPD participants (SPD_all_) with participants without SPD (AD_only_ & HC). Planned contrast were calculated merging the SPD_only_ and AD+SPD group (SPD_all_, *n* = 193). The difference in urge-to-pick-self ratings for pick-related vs. itch-related and neutral pictures was significantly higher in SPD_all_ participants compared to participants without SPD (AD_only_ & HC), with a mean difference of 1.59 (*SE* = .29, *p* = .001, $\eta_{p}^{2}$ = .047). Likewise, the difference in urge-to-pick-self ratings for picking-related vs. neutral stimuli as well as for picking-related vs. itch-related stimuli was significantly higher in SPD_all_ participants compared to participants without SPD (AD_only_ & HC), with a mean difference of 1.11 (*SE* = .18, *p* < .001, $\eta_{p}^{2}$ = .062) and .48 (*SE* = .16, *p* = .003, $\eta_{p}^{2}$ = .015). When comparing urge-to-pick-self ratings from participants with SPD_only_ to individuals affected by both AD and SPD, the difference between picking-related vs. itch-related and neutral stimuli is significantly larger for the SPD_only_ group, with a mean difference of 1.28 (*SE* = .22, *p* < .001, $\eta_{p}^{2}$ = .055). For the group comparison SPD_only_ vs. SPD+AD the difference in urge-to-pick-self ratings between picking-related and neutral stimuli as well as between picking-related and itch-related stimuli was significantly higher for SPD_only_ with a mean difference of .62 (*SE* = .13, *p* < .001, $\eta_{p}^{2}$ = .037) and .66 (*SE* = .12, p < .001, $\eta_{p}^{2}$ = .050).

#### Itch-Self Ratings

**Figure 2:**
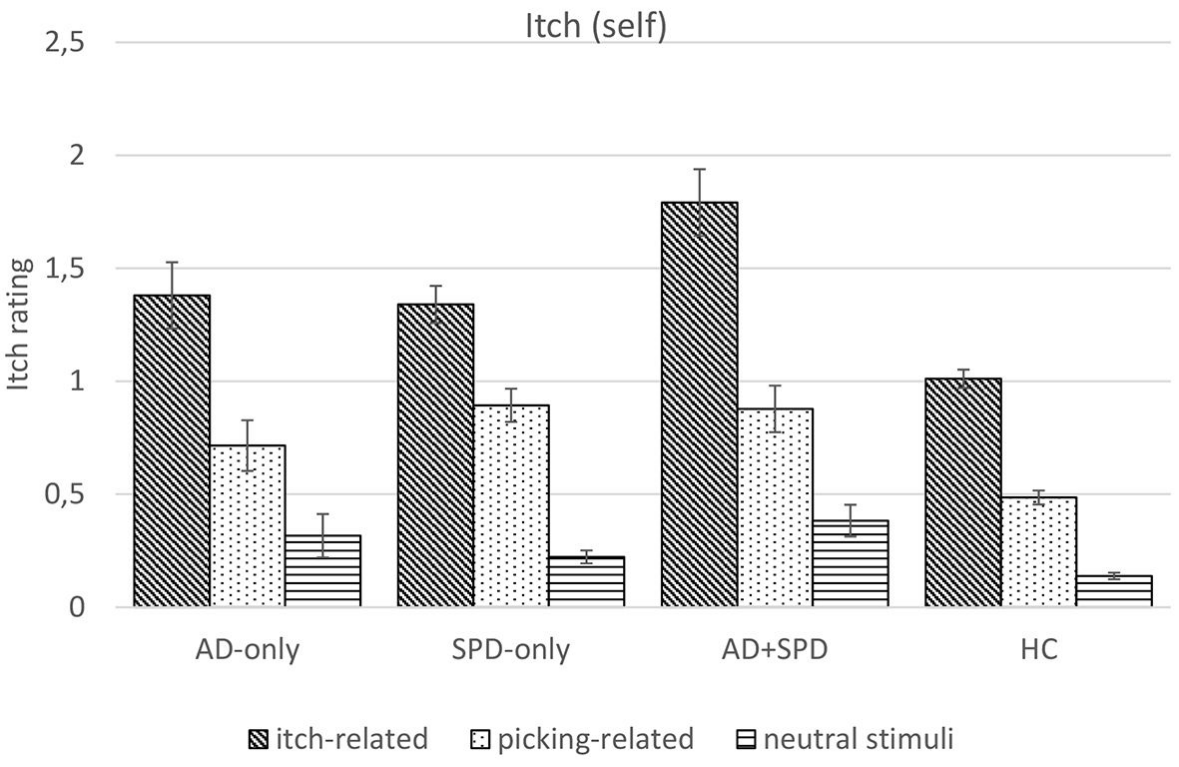
Experienced Itch (0-4) by Group and Type of Stimulus *Note.* SPD_only_ = Skin Picking Disorder; AD_only_ = Atopic Dermatitis; AD+SPD = Atopic Dermatitis and Skin Picking Disorder; HC = Healthy control. Error bars show standard errors.

There was a significant main effect on itch-self ratings for stimulus type, *F*(1.58, 940.90) = 391.95, *p* < .001, $\eta_{p}^{2}$ = .396, for group, *F*(3, 597) = 14.17, *p* < .001, $\eta_{p}^{2}$ = .066) and a significant interaction effect for stimulus type ☓ group, *F*(4.73, 940.90) = 8.06, *p* < .001, $\eta_{p}^{2}$ = .039 (see Figure 2).

All participants experienced stronger itch-sensations when looking at itch-related compared to neutral stimuli, *F*(1, 597) = 518.73, *p* < .001, $\eta_{p}^{2}$ = .465. They also reported stronger itch-sensations when looking at itch-related compared to picking-related stimuli, *F*(1, 597) = 293.72, *p* < .001, $\eta_{p}^{2}$ = .330 and when looking at picking-related compared to neutral stimuli, *F*(1, 597) = 225.76, *p* < .001, $\eta_{p}^{2}$ = .274.

To check whether participants with AD reported greater perception of itch for itch-related versus other stimuli, we compared this difference in participants with versus without AD. Planned contrast were calculated merging the AD_only_ and AD+SPD group (AD_all_, *n* = 93). The difference in itch-self ratings for itch-related vs. picking-related and neutral stimuli was significantly higher in AD_all_ compared to without AD participants (SPD_only_& HC), with a mean difference of 1.09 (*SE* = .32, *p* = .001, $\eta_{p}^{2}$ = .019).

Likewise, the difference in itch-self ratings for itch-related vs. neutral stimuli as well as for itch-related vs. picking-related stimuli was significantly higher in AD_all_ compared to without AD participants (SPD_only_ & HC), with a mean difference of .48 (*SE* = .20, *p* = .014, $\eta_{p}^{2}$ = .010) and .61 (*SE* = .15, *p* < .001, $\eta_{p}^{2}$ = .027).

## Discussion

In the presented study, we investigated whether picking-related visual stimuli trigger the urge to pick in individuals affected by SPD compared to persons without SPD. Correspondingly, we tested, whether itch-related visual stimuli trigger itch sensations in individuals with AD versus without AD. Analog to the visual provocation of itch, we demonstrated that the urge to pick can also be triggered by visual stimuli. All participants experienced a stronger urge to pick looking at pictures with picking-related content compared to neutral stimuli. Furthermore, individuals with self-reported SPD_all_ reported a significantly stronger urge to pick when looking at these stimuli compared to the AD- and HC-group. Interestingly, the SPD_only_ group showed a significantly stronger reaction to picking-related stimuli than the participants with both AD and SPD. At the same time, the AD+SPD group reported more itch-sensations to itch-related stimuli compared to the AD_only_ group. Thus, for the comorbid group the transmission of the urge to pick was less prominent than the transmission of itch-sensations. Note that the burden of skin picking as measured by the SPIS-D was higher in the SPD_only_ group (*M* = 10.59, *SD* = 4.19) compared to the comorbid group (*M* = 6.85, *SD* = 4.60). On the other hand, the psychosocial impairment due to AD (SPIS-D-AD) was higher in the comorbid group (*M* = 8.41, *SD* = 4.55) compared to the AD_only_ group (*M* = 6.36, *SD* = 4.96). The combination of SPD with comorbid AD regarding visual symptom provocation clearly requires further investigation. Even though we disseminated the survey contacting many AD specific associations, online-groups and forums, it was difficult to acquire a larger AD-sample, which limits the generalizability of our results.

This evidence for visual transmission for the urge to pick supports SPD affected self-report of different visual cues acting as triggers for picking episodes (Bohne et al., 2002; Neziroglu et al., 2008; Odlaug & Grant, 2008). The results of our study document that visual stimuli may trigger specific experiences of somatosensory perception (itch and/or the urge to pick) in the absence of somatosensory stimulation.

Not surprisingly, we were also able to replicate visual transmission of itch (Niemeier et al., 2000; Ogden & Zoukas, 2009; Papoiu et al., 2011) with people reporting to experience more itch when looking at itch-related pictures compared to other pictures (neutral, picking-related pictures). This effect was stronger for AD patients, who reported more self-itch when exposed to itch-associated skin pictures. This is again in line with previous findings on people suffering from a skin condition like AD to be more prone to visual transmission of itch than healthy controls (Papoiu et al., 2011; Schut et al., 2015).

When comparing transmission of itchiness with transmission of the urge to pick, overlapping concepts for the urge to scratch itchy skin vs. the urge to pick may be a problem. In the present sample, picking-related pictures gained significantly higher ratings for itch experience compared to neutral pictures. By presenting a short explanation including synonyms and an explanation of differences between picking and scratching, we tried to minimize the influence of this possible overlap effect. Likewise, stimuli may trigger both sensations at the same time. Furthermore, differentiating between the urge to scratch and pick may be even harder for people with both conditions (SPD and AD). Another limitation is that allocation to one of the four groups was conducted through self-report information and could not be validated by a clinician. There may have been be false-positive allocations to SPD and/or AD and conclusions on treatment of the two diagnoses need to be considered carefully. Overall, the AD_only_ sample and AD+SPD sample were underrepresented. Also, the self-reports on itch and urge to pick perception were not compared to behavioral measures such as actual scratching or picking and the urges to itch or scratch elicited were only on an average level. Finally, we recruited only female participants. Consequently, implementation objectivity, sample representativeness and external validity may be somewhat limited.

This is the first study to compare the effects of different visual stimuli as triggers for SPD compared to AD and healthy controls. Understanding the role of visual triggers for picking and/or itch episodes may help to improve treatment for both AD and SPD. In a meta-analytic review looking at efficacy of treatments for SPD (Schumer, Bartley, & Bloch, 2016) cognitive behavioral therapy (CBT) and habit reversal training (HRT) were highlighted as efficacious treatments compared to waiting list and pharmacological treatment. CBT/HRT includes assessment of picking behavior, psychoeducation, and strategies to reduce picking (e.g., HRT, relapse prevention). While HRT is a strategy designed for dealing with the overwhelming need to pick, stimulus control can be used to avoid typical trigger situations. Within stimulus control treatment, triggers are identified and then changed to reduce picking behavior (e.g., dimming the lights in the bathroom when standing in front of the mirror). This serves to strengthen alternative non-harmful behaviors. With this strategy individual visual trigger situations can be targeted specifically to prevent formation of an urge to pick (e.g., covering with clothing, limited mirror-time). Behavioral therapy for AD includes similar modules to SPD treatment. Among others, they also include techniques to reduce scratching, like HRT and stimulus control techniques (Scholz, 1999).

Further research on the transmission of itch and the urge to pick should consequently include additional (i.e., behavioral) measures for diagnoses and explore possible gender differences. For example, it would be helpful to check if the urge to pick induced by visual stimuli actually translates into picking episodes, which could be assessed in a laboratory setting. Given that most AD patients report tactile triggers for their scratching rather than visual triggers, it may be also interesting to examine the sensation of touch in absence of a tactile stimulus in these two groups. This could be accomplished, by using the somatic signal detection task (SSDT; Lloyd et al., 2008). The SSDT allows studying perceptual processes related to physical symptoms by provoking illusory tactile experiences. The number of such illusory tactile experiences may be associated with symptom severity in both AD and SPD patients.

Within this online study, the transmission of itch and the urge to pick and scratch for those effected by SPD and/or AD could be elicited using visual stimuli. The transmission of the urge to pick can serve to guide the development and improvement of interventions developed to treat SPD in the future. The present findings help to understand the relevance of visual triggers for itch/scratch and picking behaviors in AD and SPD, respectively. Looking more closely at visual triggers will aid therapists when attempting to improve treatment components targeting the onset of skin picking episodes (e.g., stimulus control techniques, HRT).

## Appendix A

**Table A1:** Urge to Pick Ratings (Self)

Sample	*n*	Stimulus type						Total	
		itch-related images		picking-related images		neutral images			
		*M*	*SD*	*M*	*SD*	*M*	*SD*	*M*	*SD*
SPD_only_	147	1.14	.95	2.00	.91	.39	.47	1.18	.65
AD_only_	47	.63	.79	.82	.82	.16	.34	.54	.58
AD+SPD	46	1.04	.87	1.25	.97	.26	.38	.85	.64
HC	361	.55	.75	.96	.86	.13	.28	.55	.55
Total	601	.74	.85	1.23	.99	.20	.37	–	–

*Note.* SPD_only_ = Skin Picking Disorder; AD_only_ = Atopic Dermatitis; AD+SPD = Atopic Dermatitis and Skin Picking Disorder; HC = Healthy control. Scale ranging from 0 (= not at all) to 4 (= very strong).

**Table A2:** Itch Ratings (Self)

Sample	*n*	Stimulus type						Total	
		itch-related images		picking-related images		neutral images			
		*M*	*SD*	*M*	*SD*	*M*	*SD*	*M*	*SD*
SP_only_	147	1.34	.99	.89	.89	.22	.35	.82	.66
AD_only_	47	1.38	1.01	.72	.77	.32	.66	.80	.72
AD+SPD	46	1.79	1.00	.88	.70	.38	.48	1.02	.63
HC	361	1.01	.96	.49	.58	.14	.28	.55	.55
Total	601	1.18	1.00	.63	.72	.19	.36	–	–

*Note.* SPD_only_ = Skin Picking Disorder; AD_only_ = Atopic Dermatitis; AD+SPD = Atopic Dermatitis and Skin Picking Disorder; HC = Healthy control. Scale ranging from 0 (= not at all) to 4 (= very strong).

## Appendix B

### B1

The 24 pictures applied as visual stimuli in this investigation were selected from a pretest with 48 pictures on a student sample (*n* = 17) to control for valence and arousal of the pictures:

In our pretest, we selected 48 pictures, four pictures of each body-part (head/face, torso/décolleté, hands/arms and legs/feet) for each of the three stimulus-types (itch-related, picking-related and neutral skin). In an online study (*n* = 17) we asked students to rate valence and arousal for each picture using the five-scale self-assessment manikin (SAM, Bradley & Lang, 1994). Out of the four pictures for each body-part category, we choose the two pictures, which had the lowest valence and arousal ratings.

The pictures will be provided by the first author upon request.

### B2

An analysis with age as covariate showed a significant main effect for age with Wilk’s Λ = .981, *F*(2, 595) = 5.86, *p* = .003, $\eta_{p}^{2}$ = .019. Including age as covariate did not relevantly change the results of the main tests as well as the post hoc tests (i.e., all previously significant results remained significant). Consequently, we decided not to include age as a covariate in the results presented.

### B3

Initial exploratory analyses revealed a few outliers. However, there was no relevant change in the pattern of results when including vs. excluding outliers. Thus, results from the complete data set are reported. The assumption of normality was not met. Since the *F*-Test is relatively robust for violation of assumption, especially in samples with more than 40 subjects, the results of the MANOVA were reported (Lindman, 1974; Tabachnick & Fidell, 2013).

### B4

**Table A3:** Short explanation to German synonyms and difference between picking and scratching (German version):

Wichtige Vorabinformation
*Skin Picking* bzw. Dermatillomanie = Erkrankung, bei der Betroffene einen starken Drang verspüren ihre Haut zu bearbeiten. Wird dem Drang nachgegangen, wird die Haut gezupft, gequetscht, an der Haut gepult und geknibbelt, was zu Hautschädigungen führen kann.
Kratzen, welches als Reaktion auf einen Neurodermitisschub erfolgt, fällt nicht unter Skin Picking.
Verwendete Synonyme im weiteren Verlauf: ***Skin Picking***, **Hautzupfen/-quetschen**, **Haut bearbeiten** und **knibbeln**.

**Table A4:** Short explanation to German synonyms and difference between picking and scratching (translated version):

Important preliminary information
*Skin Picking* or Dermatillomania = Disorder, in which affected persons feel a strong urge to manipulate their skin. If a person succumbs to that urge, the skin is plucked, squeezed, nibbled at and skin parts are removed, which can lead to skin damage.
Scratching, which occurs as a reaction to an episode of neurodermatitis, does not fall under skin picking.
Used synonyms in the further course: ***Skin picking,* skin plucking/squeezing, skin manipulation and nibbling.**

### B5

Data collection for the research reported in this study started in December 2016. Unfortunately, the local ethics committee responsible for our faculty, at that time, had not yet started to accept research proposals. The first opportunity to apply for ethical review was not possible until May 2018. Given that the study was self-funded and no funds were available for outside ethical review, it was decided to follow the procedure commonly used at that time according to the ethical guidelines of the German Psychological Society. The following procedures were included in the implementation of the study: Research participants were provided with adequate and complete information regarding participation, followed by informed consent. Specifically, all participants were informed in advance of the type of pictures they were about to see and advised on possible reactions these pictures may elicit (itch, urge to pick, some disgust). Furthermore, all participants were advised that participation was voluntary and that participants were free to end participation at any time without having to give any reasons and without having to worry about any negative consequences. Furthermore, data was assessed anonymously and participants were advised in this regard. After having received this information, all participants provided informed consent prior to participation in the study.
